# To: The Epimed Monitor ICU Database^®^: a cloud-based national registry for adult intensive care unit patients in Brazil

**DOI:** 10.5935/0103-507X.20180031

**Published:** 2018

**Authors:** Rashan Haniffa, Ambepitjwaduge Pubudu de Silva, Abigail Beane, Ponsuge Chathurani Sigera, Priyantha Lakmini Athapattu, Shriyananda Rathnayake, Kosala Saroj Amarasiri Jayasinghe, Nicolette F. de Keizer, Arjen M. Dondorp

**Affiliations:** Network for Improving Critical Care Systems and Training - Colombo, Sri Lanka; Mahidol Oxford Tropical Medicine Research Unit - Bangkok, Thailand; University of Oxford - United Kingdom of Great Britain and Northern Ireland.; Network for Improving Critical Care Systems and Training - Colombo, Sri Lanka; Ministry of Health - Colombo, Sri Lanka.; Network for Improving Critical Care Systems and Training - Colombo, Sri Lanka.; Ministry of Health - Colombo, Sri Lanka.; Information and Communication Technology Agency - Colombo, Sri Lanka.; University of Colombo Faculty of Medicine - Anuja Unnathie Abayadeera - Faculty of Medicine, University of Colombo - Colombo, Western Sri Lanka.; AMC - Medical Informatics - Amsterdam, Netherlands; National Intensive Care Evaluation Foundation - Amsterdam, Netherlands.; Mahidol Oxford Tropical Medicine Research Unit - Bangkok, Thailand; University of Oxford - United Kingdom of Great Britain and Northern Ireland.

**To the Editor**

We congratulate the Epimed collaborators^(^^1^^)^ on their impressive results from a privately owned registry in Brazil, an upper-middle income country. In addition to the examples from high income countries cited by the authors, Sri Lanka - a lower-middle-income country in South Asia - has implemented a national cloud-based intensive care unit (ICU) registry,^(^^2^^)^ directly overseen by the Ministry of Health and Information and Communications Technology Agency (ICTA), in partnership with other national and overseas collaborators, including the Dutch National Intensive Care Evaluation (NICE) foundation. Founded in 2012, the cloud-based critical care unit registry, as part of a codesigned agile mobile data platform, the so called Network for Improving Critical Care Systems and Training (NICST; www.nicst.com), encompasses almost the entire network of state ICUs island-wide and includes pediatric, neonatal and specialized units.

The national cloud-based ICU registry has many similarities to Epimed: it is prospective, web-based, and uses an international system for diagnostic coding (APACHE IV reasons for admission). This registry also enables benchmarking and facilitates research; prognostic model validation, ICU experiences of survivors, outcomes after traumatic brain injury and participation in international multi-center research projects on ventilation are some examples. The registry has highlighted challenges in the application of prognostic models such as APACHE II in this setting^(^^3^^)^ due to missing data (measurements or investigations being not performed); alternative approaches with a focus on more readily available measures have been proposed.^(^^4^^)^ In addition, perhaps uniquely for a critical care registry, national cloud-based ICU registry operates a 24/7 national critical care bed availability system that has directly assisted in locating critical care beds for over 4,500 patients.

The NICST methodology and infrastructure have been adopted in establishing national registries in Sri Lanka for renal dialysis and transplant, animal bites, cardiology, mental health and post-laparotomy patients. These registries, as part of the NICST platform, seek to utilize clinical data to directly enhance frontline clinical care while simultaneously enabling high-quality research, training and benchmarking.^(^^5^^)^ For instance, recognizing the importance of early detection of deteriorating ward patients, a parsimonious early warning score has been implemented using a mobile application (PROTECT app) as an extension of the same platform and has recorded over 500,000 observation episodes.

The utility of the NICST platform is now being evaluated in settings beyond Sri Lanka. The critical care registry is being rolled out in Pakistan in collaboration with local clinicians, and the PROTECT app has been tested for implementation in Sierra Leone.

We concur with the authors that such platforms can enable the conduct of high-quality, prospective research projects across diverse settings to evaluate the impact of variations in case-mix, resources, staffing and culture. Harnessing the power of registries in collaborative research, an often-neglected approach in non- high income countries, can enable clinicians with similar ambitions to improve the provision of acute care internationally. We look forward to working collaboratively with the authors towards these shared goals.

*Rashan Haniffa* *Network for Improving Critical Care Systems and Training - Colombo, Sri
Lanka; Mahidol Oxford Tropical Medicine Research Unit - Bangkok, Thailand;
University of Oxford - United Kingdom of Great Britain and Northern
Ireland.* *Ambepitjwaduge Pubudu de Silva* *Network for Improving Critical Care Systems and Training - Colombo, Sri
Lanka; Ministry of Health - Colombo, Sri Lanka.* *Abigail Beane* *Network for Improving Critical Care Systems and Training - Colombo, Sri
Lanka.* *Ponsuge Chathurani Sigera* *Network for Improving Critical Care Systems and Training - Colombo, Sri
Lanka.* *Priyantha Lakmini Athapattu* *Ministry of Health - Colombo, Sri Lanka.* *Shriyananda Rathnayake* *Information and Communication Technology Agency - Colombo, Sri
Lanka.* *Kosala Saroj Amarasiri Jayasinghe* *University of Colombo Faculty of Medicine - Anuja Unnathie Abayadeera -
Faculty of Medicine, University of Colombo - Colombo, Western Sri
Lanka.* *Nicolette F. de Keizer* *AMC - Medical Informatics - Amsterdam, Netherlands; National Intensive Care
Evaluation Foundation - Amsterdam, Netherlands.* *Arjen M. Dondorp* *Mahidol Oxford Tropical Medicine Research Unit - Bangkok, Thailand;
University of Oxford - United Kingdom of Great Britain and Northern
Ireland.*
